# Effects of Resistance Training on Systolic and Diastolic Blood Pressure in Hypertensive Adults: A Systematic Review and Meta-Analysis of Randomized Controlled Trials

**DOI:** 10.3390/jcm15145531

**Published:** 2026-07-15

**Authors:** Luis Romero-Vera, Julieta Lara-De la Fuente, Francisco Guede-Rojas, Claudio Carvajal-Parodi, Sergio Araya-Sierralta, Jorge Pérez-Contreras, David Ulloa-Díaz, Ángela Rodríguez-Perea

**Affiliations:** 1 Facultad de Salud y Ciencias Sociales, Escuela de Ciencias de la Actividad Física, Universidad de Las Américas, Concepción 4030000, Chile; luis.romero.vera@edu.udla.cl; 2 Pedagogía en Educación Física, Facultad de Educación, Universidad Católica de la Santísima Concepción, Cocepción 4030000, Chile; jlara@educfisica.ucsc.cl; 3 Exercise and Rehabilitation Science Institute, School of Physical Therapy, Faculty of Rehabilitation Science, Universidad Andres Bello, Santiago 7591538, Chile; francisco.guede@unab.cl; 4 Escuela de Kinesiología, Facultad de Ciencias de la Rehabilitación y Calidad de Vida, Universidad San Sebastián, Concepción 4030000, Chile; claudio.carvajal@uss.cl; 5 Facultad de Humanidades y Educación, Universidad de Atacama, Copiapó 1531772, Chile; 6 Escuela de Ciencias del Deporte y Actividad Física, Facultad de Salud, Universidad Santo Tomas, Santiago 8370000, Chile; joperezc@gmail.com; 7 Magíster en Evaluación y Planificación del Entrenamiento Deportivo, Facultad de Ciencias de la Vida, Universidad Viña del Mar, Viña del Mar 2520000, Chile; 8 Department of Sports Sciences and Physical Conditioning, Universidad Católica de la San Tísima Concepción, Concepción 4030000, Chile; 9 Department of Physical Education and Sport, Faculty of Physical Activity and Sports Sciences, Universidad de León, 24007 León, Spain; arodrp@unileon.es; 10 Strength & Conditioning Laboratory, CTS-642 Research Group, Department of Physical Education and Sport, Faculty of Sport Sciences, University of Granada, 18011 Granada, Spain

**Keywords:** hypertension, high blood pressure, systolic blood pressure, diastolic blood pressure, resistance training, strength training, randomized controlled trials, meta-analysis

## Abstract

**Background and Objectives**: Hypertension is an important clinical condition associated with increased cardiovascular morbidity and mortality. Resistance training has been proposed as a non-pharmacological strategy for blood pressure control; however, its clinical relevance in hypertensive adults remains unclear because of methodological heterogeneity and differences in training prescription. This systematic review and meta-analysis aimed to analyse the effects of resistance training on systolic blood pressure (SBP) and diastolic blood pressure (DBP) in adults with hypertension or elevated blood pressure, with emphasis on the magnitude of the effects and a cautious interpretation of their clinical relevance. **Materials and Methods**: A systematic search was conducted in Scopus, PubMed, EBSCOhost and Web of Science from database inception to 1 June 2026. Randomised controlled trials were eligible if they included adults with hypertension or elevated blood pressure, examined resistance training interventions, and reported SBP and DBP values before and after the intervention. Twelve randomised controlled trials were included. Standardised mean differences (SMDs) with 95% confidence intervals (CIs) were calculated using inverse-variance random-effects models. **Results**: Resistance training produced a significant reduction in SBP compared with control or comparator groups, with a moderate pooled effect (SMD = −0.77; 95% CI: −1.06 to −0.48; *p* < 0.00001; I^2^ = 35%). DBP also decreased significantly, with a smaller pooled effect (SMD = −0.43; 95% CI: −0.67 to −0.19; *p* = 0.0003; I^2^ = 0%). **Conclusions**: Resistance training reduces SBP and DBP in adults with hypertension or elevated blood pressure. Its clinical application should be considered complementary to pharmacological treatment. Because individual participant data and categorical transition analyses were not available, the current evidence does not confirm systematic reclassification across AHA/ACC blood pressure categories. Standardised protocols and analyses based on absolute blood pressure changes in mmHg are required.

## 1. Introduction

Hypertension (HTN) is a clinical condition that increases the risk of cardiovascular disease, stroke, heart failure, chronic kidney disease, and premature mortality through progressive damage to the vascular system and target organs [1,2,3,4]. Systolic and diastolic blood pressure are continuously associated with cardiometabolic risk, even below diagnostic thresholds [5,6,7,8]. The high global prevalence of HTN, suboptimal therapeutic control, and the increasing burden among young and middle-aged adults support the need for complementary prevention and treatment strategies [5,6,9,10]. Pharmacological treatment remains the cornerstone of clinical management, particularly in patients with established HTN or high cardiovascular risk [1,2,4,7]. However, effective management also depends on adherence, individual response, comorbidities, sustained follow-up and the integration of non-pharmacological strategies, including dietary improvement, sodium reduction, body weight control, reduced sedentary behaviour and physical exercise [1,2,4,8,9,10,11].

Physical exercise is a cost-effective non-pharmacological strategy for the prevention and treatment of HTN because it can reduce blood pressure in individuals with elevated blood pressure or established hypertension [11,12,13,14]. Continuous aerobic training has the strongest historical support, with favourable effects on systolic and diastolic blood pressure, ambulatory blood pressure, endothelial function and cardiovascular risk [12,13,14,15]. These effects are partly explained by improvements in nitric oxide bioavailability, autonomic regulation, vascular function and peripheral vascular resistance [12,16,17,18]. Other exercise modalities, including high-intensity interval training and low-intensity interval training, have also shown antihypertensive effects, although their clinical magnitude may vary across outcomes, adherence levels, baseline cardiovascular risk and population characteristics [14,15,19]. Therefore, comparing aerobic, interval, combined and resistance-based interventions is clinically relevant for identifying safe, effective and applicable exercise strategies in hypertensive adults [11,12,13,14,15,20].

Resistance training (RT) has an increasingly important role in exercise recommendations for both clinical and non-clinical populations [11,20,21]. In patients with HTN, RT is recognised as a complementary strategy that may reduce blood pressure, improve muscular strength, optimise physical function and contribute to cardiometabolic control [17,18,21,22]. In older and clinically vulnerable adults, gains in muscle strength are relevant beyond blood pressure control because they may improve functional independence, balance, mobility and the capacity to perform daily activities and may contribute to a lower risk of falls when RT is appropriately prescribed and supervised [20,21,22,23]. RT includes dynamic, isometric, elastic band-based, machine-based, free-weight, bodyweight and combined protocols involving different muscle actions [17,20,22,24]. Dynamic RT may induce musculoskeletal, metabolic and vascular adaptations, including improvements in muscle mass, metabolic sensitivity, endothelial function and functional capacity [20,21,22,23]. Isometric training, particularly handgrip training, has also shown reductions in blood pressure in clinical trials and meta-analyses, although the magnitude of the effect varies between protocols [19,20,24,25].

Recent systematic reviews and meta-analyses report that RT may reduce blood pressure in adults with elevated blood pressure, prehypertension or HTN [17,18,19,20]. Potential mechanisms include improved endothelial function, increased nitric oxide bioavailability, reduced arterial stiffness, lower sympathetic activity, enhanced baroreflex sensitivity and favourable changes in body composition [21,22,23,26]. The response to RT appears to depend on supervision, medication status, baseline blood pressure, type of contraction, muscle mass involved, programme duration, volume and intensity [17,18,20,22]. Interindividual variability is clinically relevant because some participants show meaningful reductions, whereas others present minimal changes or no response [22,24,27,28]. In addition, heterogeneity in intervention protocols, populations, measurement procedures, and pharmacological control limits reproducibility and prevents the inference of a single prescription model [17,18,22,24]. The clinical relevance of RT-induced changes in blood pressure categories remains insufficiently explored, supporting the need for an updated synthesis focused on hypertensive adults [17,18,19,20,29,30].

RT protocols differ not only by exercise modality but also by the way the training stimulus is prescribed and progressed. Key load variables include weekly frequency, exercise selection, session duration, contraction type, rest interval, volume, intensity, intensity-control method and progression criteria [19,21,22,23,24,25]. These variables may influence haemodynamic, vascular, autonomic and neuromuscular adaptations. Therefore, the clinical interpretation of RT in adults with HTN should consider both the modality used and the specific characteristics of the training dose [19,20,21,22,23,24,25].

Previous systematic reviews and meta-analyses have grouped intervention protocols by exercise modality or focused on overall antihypertensive effects. However, randomised controlled trials of RT describing training-load variables and their effects on clinical blood pressure categories have not been sufficiently examined [13,15,16,17,18,29,30]. Therefore, this systematic review and meta-analysis aimed to evaluate the effect of RT on SBP and DBP in adults with HTN or elevated blood pressure. The specific objectives were: (I) to evaluate the evidence on the effects of RT interventions on SBP and DBP; (II) to determine whether these effects differed according to RT modality; and (III) to discuss the clinical relevance and limitations of these effects without assuming individual diagnostic reclassification when categorical transition data were unavailable.

## 2. Materials and Methods

### 2.1. Study Design

**Figure 1:**
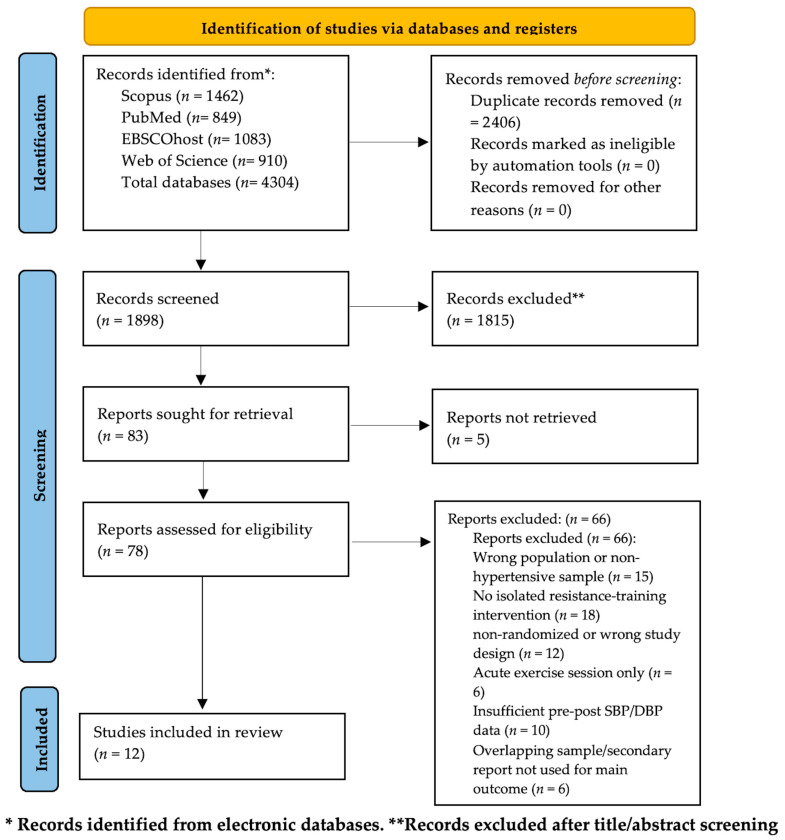
Preferred Reporting Items for Systematic Reviews and Meta-Analyses flow diagram, showing the article selection process.

This systematic review and meta-analysis was conducted and reported in accordance with the Preferred Reporting Items for Systematic Reviews and Meta-Analyses 2020 statement (PRISMA 2020, Ottawa, ON, Canada) [31]. The PRISMA 2020 checklist was completed and is provided as Supplementary Table S1. The study selection process was summarised using the PRISMA 2020 flow diagram (Figure 1). The review protocol was prospectively registered in the International Prospective Register of Systematic Reviews (PROSPERO, United Kingdon, England) under registration number CRD420261386510.

The protocol specified the population, intervention, comparator, outcomes, eligibility criteria, search strategy, risk-of-bias assessment, and planned quantitative synthesis. No major deviations from the registered protocol were introduced during the review process; post hoc clarifications were limited to the separate interpretation of clinical/office and ambulatory blood pressure outcomes and to the qualitative handling of overlapping or non-isolable intervention arms.

### 2.2. Search Strategy

Systematic searches were conducted in four electronic databases: Scopus, PubMed, EBSCOhost, and Web of Science, covering all records from database inception to 1 June 2026. Controlled vocabulary and free-text terms related to hypertension and resistance training were combined using Boolean operators. Duplicate records were removed, and two independent reviewers screened the titles, abstracts, and full texts of articles written in English. 

Terms related to hypertension were: “Hypertension”, “High Blood Pressure”, “Arterial Hypertension”, “Primary Hypertension” and “Blood Pressure, High”.

Terms related to resistance training were: “Resistance Training”, “Training, Resistance”, “Strength Training”, “Training, Strength”, “Dynamic Resistance Training”, “Isometric Training”, “Isometric Handgrip”, “Elastic Band Training” and “Strength Exercise”. The complete search strategy for each database is provided in Table 1. The core search strategy used across all databases was as follows:

((((((((Hypertension) OR (“High Blood Pressure”)) OR (“Arterial Hypertension”)) OR (“Primary Hypertension”)) OR (“Blood Pressure, High”)) AND (“Resistance Training”)) OR (“Training, Resistance”)) OR (“Strength Training”)) OR (“Training, Strength”)

### 2.3. Eligibility Criteria

Studies were included if they met the following eligibility criteria: (i) adults aged ≥18 years with diagnosed hypertension or elevated blood pressure, including prehypertension/elevated blood pressure when explicitly reported by the original study; (ii) a chronic RT intervention lasting at least six weeks, including dynamic RT, isometric handgrip training, elastic band-based RT or combined protocols with an identifiable RT component; (iii) comparison with a non-exercise control group, usual care, or another exercise intervention; (iv) available SBP and DBP values before and after the intervention, or sufficient information to derive these values; and (v) a randomised controlled trial design. Blood pressure categories were interpreted according to the 2025 American Heart Association/American College of Cardiology (AHA/ACC) classification [32]: normal blood pressure was defined as <120/<80 mmHg; elevated blood pressure as SBP 120–129 mmHg with DBP <80 mmHg; stage 1 hypertension as SBP 130–139 mmHg or DBP 80–89 mmHg; stage 2 hypertension as SBP ≥140 mmHg or DBP ≥ 90 mmHg; and severe hypertension/hypertensive crisis as values above 180 mmHg and/or 120 mmHg.

Studies were excluded if: (i) the intervention consisted only of a single acute exercise session; (ii) the study used a non-randomised design; (iii) participants were athletes, exclusively normotensive samples or healthy young adults without hypertension or elevated blood pressure; (iv) the intervention did not include an RT component that could be isolated for qualitative or quantitative interpretation; (v) the chronic effects of RT could not be separated from another intervention component; (vi) the study lacked blood pressure data before and after the intervention or sufficient information to estimate these values; or (vii) the article was not published in English.

For the quantitative synthesis, studies were grouped according to the resistance training modality described in the intervention arm: dynamic resistance training, isometric handgrip training, elastic band-based resistance training, and combined resistance training protocols. Clinical/office blood pressure and 24 h ambulatory blood pressure were not pooled as equivalent outcomes because they differ in measurement procedures and clinical interpretation. When multiple eligible resistance training arms shared a single comparator, the comparator sample size was divided across comparisons to avoid double counting.

### 2.4. Data Extraction

Two independent reviewers extracted the following information from the eligible studies: (i) study characteristics, including title, authors, year of publication, and country; (ii) participant characteristics, including sample size, sex, age, hypertension status, and medication use; (iii) comparator characteristics; (iv) SBP and DBP values before and after the intervention; (v) pharmacological control procedures; and (vi) interventions, which were descriptively examined according to key training-load variables, including intervention duration, weekly frequency, type of RT, intervention description (sets, repetitions and exercises), intensity control and intensity manipulation. These variables were extracted to provide a more detailed interpretation of the training stimulus and to identify whether the available evidence allowed a clear dose-response interpretation. The extracted data were checked between reviewers. Discrepancies were resolved by consensus, and effect sizes were calculated from the available data when they were not reported by the original study.

### 2.5. Risk of Bias Assessment

The risk of bias of each study was independently assessed by two reviewers (G.M. and F.G.) using the revised Cochrane risk-of-bias tool for randomised trials (RoB 2) [33]. The official RoB 2 domains were evaluated: D1, bias arising from the randomisation process; D2, bias due to deviations from the intended interventions; D3, bias due to missing outcome data; D4, bias in measurement of the outcome; and D5, bias in selection of the reported result. Each domain was judged as low risk of bias, having some concerns, or high risk of bias according to the signalling questions and the RoB 2 algorithm. The overall risk of bias was derived from the domain-level judgements. Discrepancies were resolved by consensus or through consultation with a third reviewer (A.R.).

### 2.6. Publication Bias

Publication bias was examined visually using Begg’s funnel plot and statistically using Begg’s rank correlation test [34] and Egger’s regression test [35]. The Duval and Tweedie “trim-and-fill” method [36] was applied to account for potential bias. Sensitivity analyses were performed to determine the robustness of the results.

### 2.7. Statistical Analyses

All calculations were performed using a Microsoft Excel spreadsheet (Microsoft, Redmond, WA, USA) containing the data extracted from each publication. Review Manager (RevMan), version 5.4.5, was used to conduct the meta-analyses and generate the forest plots. The primary effect measure was the standardised mean difference (SMD) with 95% confidence interval (CI), calculated using the inverse-variance method and a DerSimonian-Laird random-effects model. A random-effects framework was selected because clinical and methodological heterogeneity was expected across RT modalities, intervention duration, intensity control, participant characteristics, pharmacological treatment and blood pressure measurement procedures.

Post-intervention values were used as the primary data source when means, SDs, and sample sizes were available for the experimental and comparator groups. Change scores were used when post-intervention values were unavailable or when the original article reported changes as the primary outcome. The SMD was used as the primary pooled metric considering the types of trials, differences in the measurement context, including clinical/office, resting and ambulatory blood pressure, the reporting format, the availability of dispersion data, and the need for imputation in several studies. Therefore, absolute changes in mmHg were considered descriptively when available, and the clinical interpretation of SMD-based results was explicitly performed with caution.

For studies with multiple resistance training arms sharing the same comparator group, the comparator sample size was divided across the relevant comparisons to avoid double counting. The mean and SD of the comparator group were kept unchanged, whereas the total sample size was divided proportionally according to the number of intervention arms included in the same analysis.

Between-study heterogeneity was assessed using Cochran’s Q statistic and the I^2^ statistic [37]. I^2^ values were interpreted as low, moderate, substantial, or high heterogeneity according to commonly used thresholds. Study-specific weights were derived from the inverse-variance method. Effect sizes were interpreted as trivial (<0.2), small (≥0.2 to <0.5), moderate (≥0.5 to <0.8) or large (≥0.8) [38].

Subgroup analyses were performed according to RT modality: dynamic RT, isometric handgrip training, elastic band-based RT and combined RT protocols. Clinical/office blood pressure and 24 h ambulatory blood pressure were analysed separately because of methodological and diagnostic differences in outcome assessment. Combined protocols that included an aerobic component were interpreted with caution because the isolated effect of RT could not be fully determined. A post hoc sensitivity interpretation was also performed by examining whether the direction and significance of the findings were maintained after excluding combined protocols containing an aerobic component; this analysis was interpreted conservatively because of the limited number of studies in each subgroup. An exploratory intensity-based interpretation was additionally considered for studies reporting %1RM or equivalent intensity markers. However, intensity was expressed using non-equivalent metrics, including %1RM, %MVC, repetition zones, RPE, and elastic resistance progression, which prevented a robust formal meta-analysis using a single intensity scale. Medication-status subgrouping was also considered, but most trials did not provide separable outcome data for medicated and non-medicated participants; therefore, medication use was handled as a clinical heterogeneity factor and interpreted descriptively.

### 2.8. Certainty of the Evidence

The certainty of the evidence for the primary outcomes (SBP and DBP) was assessed using the Grading of Recommendations Assessment, Development and Evaluation (GRADE) approach [39]. Evidence from randomised controlled trials was initially considered to be of high certainty and was downgraded when concerns were identified regarding risk of bias, inconsistency, indirectness, imprecision or publication bias. Certainty was categorised as high, moderate, low or very low.

## 3. Results

### 3.1. Study Selection

The database search identified 4304 records across Scopus (*n* = 1462), PubMed (*n* = 849), EBSCOhost (*n* = 1083) and Web of Science (*n* = 910). After removing 2406 duplicate records, 1898 records were screened by title and abstract. Of these, 1815 records were excluded because they did not meet the predefined eligibility criteria. Eighty-three reports were sought for retrieval, of which five could not be retrieved. Therefore, 78 full-text reports were assessed for eligibility. Sixty-six reports were excluded for the following reasons: wrong population or non-hypertensive sample (*n* = 14), no isolated resistance-training intervention (*n* = 8), non-randomised or wrong study design (*n* = 12), acute exercise session only (*n* = 6), insufficient pre-post SBP/DBP data (*n* = 19), and overlapping sample or secondary report not used for the main outcome (*n* = 6). Finally, 12 [40,41,42,43,44,45,46,47,48,49,50,51] randomised controlled trials were included in the systematic review and meta-analysis.

### 3.2. Study Characteristics

The included randomised controlled trials examined different types of RT in adults with hypertension or high blood pressure [21,22,24,40,41,42,43,44,45,46,47,48]. The interventions included dynamic strength training [21,22,24,40,41,42,46], isometric handgrip training [21,43,47], elastic band training [44] and combined strength/resistance training protocols [21,22,46,48]. The duration of the interventions ranged from 6 to 12 weeks, with a predominant frequency of two to four sessions per week [21,22,24,40,41,42,43,44,45,46,47,48]. Systolic and diastolic blood pressure were the primary outcomes extracted for the meta-analysis [21,22,24,40,41,42,43,44,46,47]. Blood pressure was assessed using clinical/office blood pressure, resting blood pressure, or 24-h ambulatory monitoring, depending on the original design of each study [21,22,24,40,41,42,43,44,45,46,47,48]. Although 12 randomised controlled trials were included, some studies provided more than one eligible comparison because they included multiple RT arms [21,22,24,41,46,47,48]. The sample sizes of the shared comparator groups were divided among the corresponding comparisons to avoid double counting [21,22,24,41,46,47,48].

With regard to pharmacological control, the included studies reported different criteria for stabilisation and monitoring of antihypertensive treatment [21,24,40,41,42,43,45,46,47,48]. Most trials kept the prescribed medication unchanged throughout the intervention period, with the aim of isolating the effect of RT on blood pressure [21,24,40,41,43,45,46,47,48]. Some studies included both medicated and unmedicated participants, whereas others restricted the sample to subjects on stable pharmacological treatment or excluded those who altered their treatment regimen during follow-up [21,24,40,41,42,43,45,46,47,48]. A separate quantitative sub-analysis of non-medicated participants was not possible because the available trials generally did not report blood pressure outcomes stratified by medication status. This heterogeneity reflects typical clinical conditions in adults with hypertension or elevated blood pressure [21,22,24,40,41,42,43,44,45,46,47,48]. The use of antihypertensive medication was considered in the interpretation of the results due to its potential influence on the magnitude of the changes observed in systolic and diastolic blood pressure [21,24,40,41,42,43,45,46,47,48].

### 3.3. Effects of Resistance Training on Systolic Blood Pressure

RT showed a consistent reduction in systolic blood pressure in most of the included comparisons. The direction of the effect favoured the experimental group, particularly in dynamic strength training protocols and in interventions involving participants with higher baseline SBP values. The clinical relevance of these changes was interpreted with caution because the pooled analysis used standardised mean differences rather than absolute reductions in mmHg. In Table 2, we show the Characteristics of the RCTs included in the current systematic review and meta-analysis.

**Figure 2:**
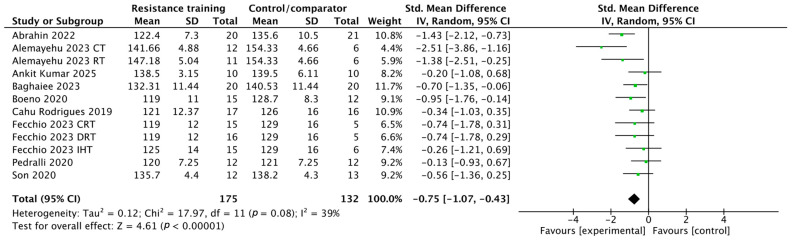
Forest plot of the effect of resistance training on systolic blood pressure. Abrahin 2022 [41], Abrahin 2024 [40], Alemayehu 2023 [46], Ankit Kuma 2025 [47], Baghaiee 2023 [42], Boeno 2020 [24], Cahu Rodrigues 2019 [43],Fecchio 2023 [21], Pedralli 2020 [22], Son 2020 [44].

The meta-analysis showed a significant reduction in systolic blood pressure in favour of RT compared with control or comparator groups. The pooled effect was SMD = −0.77; 95% CI: −1.06 to −0.48; Z = 5.28; *p* < 0.00001. Heterogeneity was low to moderate, with Tau^2^ = 0.09; Chi^2^ = 18.33; df = 12; *p* = 0.11; I^2^ = 35%. The corresponding forest plot is shown in Figure 2.

### 3.4. Effects of Resistance Training on Diastolic Blood Pressure

The effects of RT on diastolic blood pressure were smaller than those observed for systolic blood pressure. Several studies showed reductions in DBP, but the magnitude of change was generally lower. DBP appeared to be less sensitive to RT interventions, particularly in participants whose baseline DBP values were closer to controlled ranges or who were receiving stable antihypertensive treatment.

**Figure 3:**
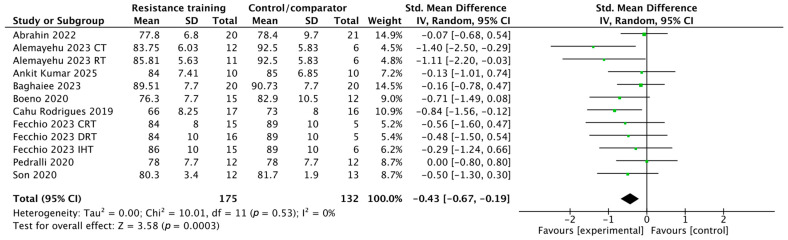
Forest plot of the effect of resistance training on diastolic blood pressure. Abrahin 2022 [41], Alemayehu 2023 [46], Ankit Kuma 2025 [47], Baghaiee 2023 [42], Boeno 2020 [24], Cahu Rodrigues 2019 [43],Fecchio 2023 [21], Pedralli 2020 [22], Son 2020 [44].

The meta-analysis showed a significant reduction in diastolic blood pressure in favour of RT compared with control or comparator groups. The pooled effect was SMD = −0.43; 95% CI: −0.67 to −0.19; Z = 3.58; *p* = 0.0003. There was no heterogeneity, with Tau^2^ = 0.00; Chi^2^ = 10.01; df = 11; *p* = 0.53; I^2^ = 0%. The corresponding forest plot is shown in Figure 3.

### 3.5. Subgroup Analysis According to Resistance Training Modality

The subgroup analysis based on training modality showed different magnitudes of effect for SBP and DBP. For SBP, the overall effect favoured RT compared with control or comparator groups (SMD = −0.76; 95% CI: −1.05 to −0.48; *p* < 0.00001). Overall heterogeneity was low to moderate (I^2^ = 35%). The test for subgroup differences was not statistically significant (χ^2^ = 6.41; df = 3; *p* = 0.09; I^2^ = 53.2%), although the direction of the estimates suggested modality-specific variability.

**Figure 4:**
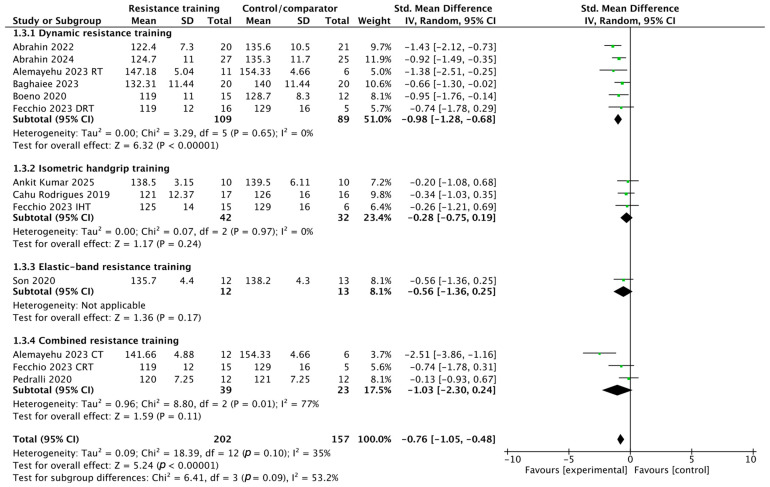
Forest plot of the effect of resistance training on systolic blood pressure according to training modality. Abrahin 2022 [41], Abrahin 2024 [40], Alemayehu 2023 [46], Baghaiee 2023 [42], Boeno 2020 [24], Fecchio 2023 [21], Ankit Kuma 2025 [47], Cahu Rodrigues 2019 [43], Son 2020 [44]., Pedralli 2020 [22].

Dynamic strength training had the most consistent effect on systolic blood pressure, with a significant reduction in favour of the intervention (SMD = −0.98; 95% CI: −1.28 to −0.68; *p* < 0.00001). This subgroup included six intervention arms, with low heterogeneity between studies (I^2^ = 0%). Isometric handgrip training showed a small, non-significant effect on systolic blood pressure (SMD = −0.28; 95% CI: −0.75 to 0.19; *p* = 0.24), also with no heterogeneity (I^2^ = 0%). Training with elastic bands showed a non-significant reduction (SMD = −0.56; 95% CI: −1.36 to 0.25; *p* = 0.17), limited by the inclusion of a single study. Combined protocols showed a large, though non-significant, effect size (SMD = −1.03; 95% CI: −2.30 to 0.24; *p* = 0.11), with considerable heterogeneity (I^2^ = 77%), indicating significant variability among the included protocols. The forest diagram corresponding to Figure 4 shows the effects of Resistance Training modalities on systolic pressure.

**Figure 5:**
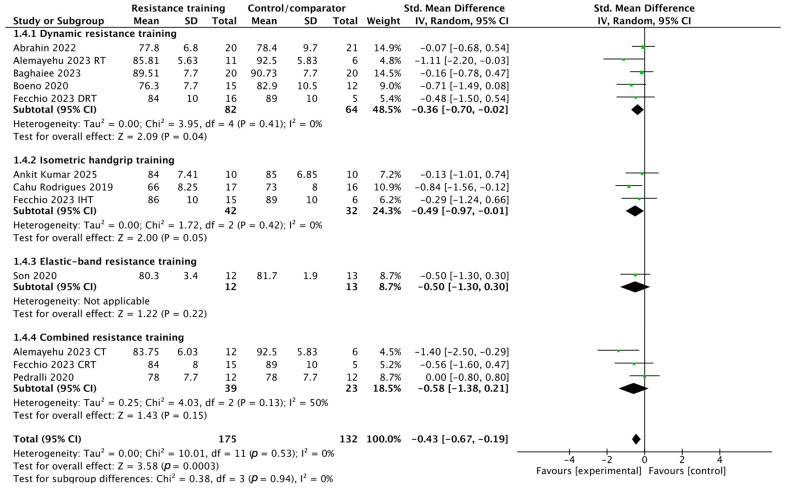
Forest plot of the effect of resistance training on diastolic blood pressure according to training modality. Abrahin 2022 [41], Alemayehu 2023 [46], Baghaiee 2023 [42], Boeno 2020 [24], Fecchio 2023 [21], Ankit Kuma 2025 [47], Cahu Rodrigues 2019 [43], Son 2020 [44]., Pedralli 2020 [22].

For DBP, the overall effect also favoured RT, with a significant small-to-moderate reduction (SMD = −0.43; 95% CI: −0.67 to −0.19; *p* = 0.0003). Overall heterogeneity was absent or very low (I2 = 0%). Dynamic strength training showed a significant reduction in DBP (SMD = −0.36; 95% CI: −0.70 to −0.03; *p* = 0.04). Isometric handgrip training also showed a significant small-to-moderate effect (SMD = −0.49; 95% CI: −0.97 to −0.01; *p* = 0.04). Elastic band training showed a non-significant reduction (SMD = −0.50; 95% CI: −1.29 to 0.30; *p* = 0.22), based on one study. Combined protocols showed a non-significant reduction in DBP (SMD = −0.59; 95% CI: −1.39 to 0.21; *p* = 0.15), with moderate heterogeneity. The forest diagram corresponding to Figure 5 shows the effects of Resistance Training modalities on diastolic pressure.

The results suggest that dynamic strength training provides the most consistent evidence for reducing SBP, whilst both dynamic training and isometric handgrip training have favourable effects on DBP. The results for the subgroups involving elastic bands and combined protocols should be interpreted with caution due to the small number of studies, methodological heterogeneity and the difficulty in isolating the specific effect of the RT component in multi-component interventions.

### 3.6. Changes in Blood Pressure According to Resistance Training Modality

Because the included trials did not report individual participant transitions across the 2025 AHA/ACC blood pressure categories [32], categorical reclassification could not be directly quantified, and the observed changes should not be interpreted as sufficient evidence of reclassification. Therefore, changes in blood pressure according to RT modality were interpreted descriptively using group-level pre- and post-intervention values. Dynamic strength training showed the most consistent systolic reductions, particularly in studies with baseline SBP values close to diagnostic thresholds. Isometric handgrip training showed more variable systolic effects, whereas combined protocols were difficult to interpret because dynamic, isometric, and aerobic components were sometimes integrated within the same intervention. DBP contributed less to potential category changes because reductions were generally smaller and several baseline DBP values were closer to controlled ranges. These findings suggest possible clinical relevance but do not demonstrate systematic diagnostic reclassification.

### 3.7. Exploratory Analysis According to Exercise Intensity and Medication Status

Exercise intensity was considered as a potential effect modifier because the included protocols used different relative loads and progression criteria. Dynamic RT protocols using moderate-to-high loads (approximately 50–85% 1RM, 8–20 repetition-maximum zones or RPE-guided progression) showed the most consistent direction of SBP reduction. Isometric handgrip protocols were usually prescribed at 30% MVC and showed smaller or less consistent systolic effects, whereas elastic-band and combined protocols were limited by single-study evidence or by the inclusion of aerobic components. However, a formal meta-analysis using a single intensity category was not statistically appropriate because intensity was not reported on a common scale across modalities. The medication-status sub-analysis requested during peer review was also limited by the absence of separable outcome data for medicated and non-medicated participants. Therefore, both intensity and medication status were interpreted as exploratory clinical modifiers rather than definitive subgroup effects.

### 3.8. Sensitivity and Methodological Considerations

The interpretation of the results considered methodological differences between studies, including the blood pressure measurement method, intervention type, intensity control, intervention duration, pharmacological management, and baseline blood pressure. Clinical/office blood pressure and 24-h ambulatory blood pressure were not pooled as equivalent outcomes because they differ in diagnostic and prognostic value. Caminiti et al. (2021) [48] was retained for the qualitative description but excluded from the main meta-analysis because the chronic effect of RT could not be isolated. Silva de Sousa et al. (2024) [45] was not pooled with Fecchio et al. (2023) [21] for clinical/office blood pressure outcomes because both publications derived from the same original trial. Therefore, Silva de Sousa et al. (2024) [45] was considered only for ambulatory blood pressure outcomes. When combined protocols containing aerobic exercise wee excluded from the interpretation, the direction of the effect remained favourable to RT, particularly for dynamic RT on SBP and for dynamic and isometric handgrip training on DBP; however, this post hoc sensitivity interpretation should be considered exploratory because of the small number of available comparisons.

**Figure 6:**
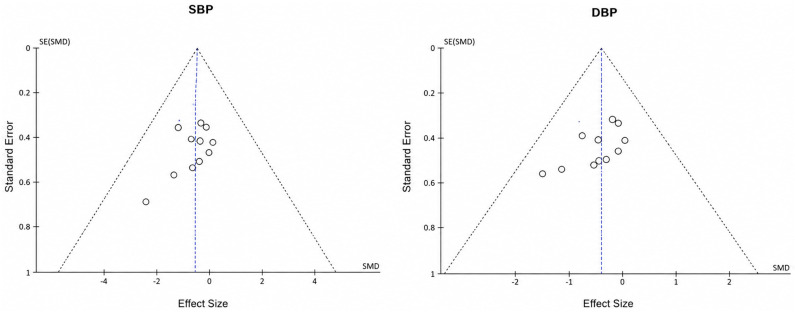
Funnel plots for the assessment of potential publication bias in studies evaluating the effects of resistance training on systolic blood pressure and diastolic blood pressure.

Publication bias was visually assessed using funnel plots for SBP and DBP. The plots showed moderate dispersion around the pooled effect estimate, with no clear evidence of marked asymmetry. However, this interpretation should be considered exploratory because funnel plots and statistical tests for small-study effects have limited reliability when the number of included studies or comparisons is small and when intervention protocols are heterogeneous. The funnel plots for SBP and DBP are presented in Figure 6.

**Figure 7:**
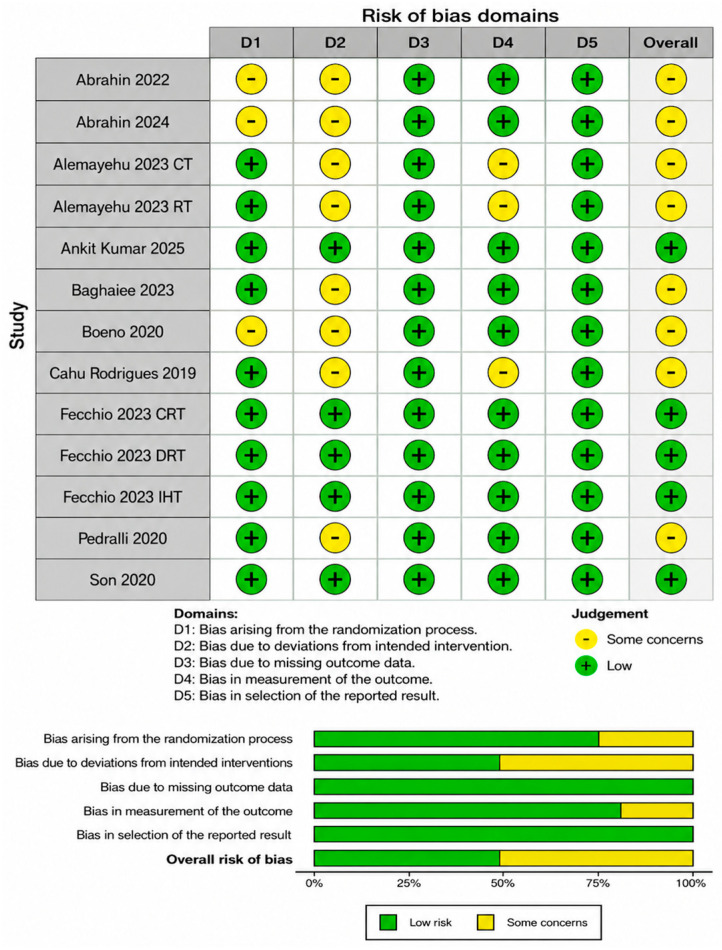
Risk-of-bias assessment of the included randomised controlled trials using the revised Cochrane risk-of-bias tool for randomised trials (RoB 2). Abrahin 2022 [41], Abrahin 2024 [40], Alemayehu 2023 [46], Ankit Kuma 2025 [47], Baghaiee 2023 [42], Boeno 2020 [24], Cahu Rodrigues 2019 [43], Fecchio 2023 [21], Pedralli 2020 [22], Son 2020 [44].

Using the RoB 2 framework, most studies showed a low risk of bias for missing outcome data (D3) and selection of the reported result (D5). Some concerns were identified mainly for bias arising from the randomisation process (D1), deviations from the intended interventions (D2) and measurement of the outcome (D4). Overall, several studies were classified as having some concerns, whereas the remaining studies showed a low overall risk of bias. No study was judged to have a high overall risk of bias. The complete RoB 2 assessment is presented in Figure 7.

### 3.9. Certainty of the Evidence

The certainty of the evidence was rated as moderate for SBP and low to moderate for DBP (Table 3). SBP was downgraded mainly because of clinical heterogeneity, imputed dispersion values in some studies, and variability in RT modality and pharmacological control. DBP was additionally downgraded because of the smaller magnitude of the effect and potential imprecision in several comparisons. No outcome was rated as high certainty because of methodological heterogeneity and incomplete reporting of absolute changes in mmHg.

## 4. Discussion

This systematic review and meta-analysis evaluated the effects of RT on SBP and DBP in adults with hypertension or elevated blood pressure. RT significantly reduced SBP, with a moderate pooled effect (SMD = −0.77; 95% CI: −1.06 to −0.48; *p* < 0.00001) and low-to-moderate heterogeneity (I^2^ = 35%). RT also reduced DBP, although the pooled effect was smaller (SMD = −0.43; 95% CI: −0.67 to −0.19; *p* = 0.0003) and heterogeneity was negligible (I^2^ = 0%). These findings support a favourable antihypertensive effect of RT, particularly for SBP. Nevertheless, clinical interpretation should remain cautious because SMDs do not provide direct pooled reductions in mmHg. Therefore, the results should be interpreted as evidence of a favourable direction and magnitude of effect, rather than as proof of systematic diagnostic reclassification according to AHA/ACC blood pressure categories [1,2,32].

The use of SMD should be recognised as a methodological limitation because blood pressure is conventionally measured, pooled and interpreted in mmHg. Although SMDs allowed quantitative synthesis under heterogeneous reporting conditions and incomplete dispersion data, they reduce direct clinical interpretability and prevent the estimation of absolute treatment effects. Accordingly, the present results describe the direction and relative magnitude of the RT effect rather than a pooled mmHg reduction.

The clinical relevance of the observed effects depends on baseline blood pressure, the absolute magnitude of change, and the proximity of each participant to diagnostic thresholds. Moderate reductions in blood pressure may be meaningful at the population level when sustained over time and integrated into multifactorial treatment [7,49,50,51,52]. However, a participant with stage 2 hypertension may remain in the same category if final values do not decrease below 140/90 mmHg [1,4,5,32]. Similarly, a participant with stage 1 hypertension may remain in the same category when DBP remains ≥80 mmHg, even if SBP decreases below 130 mmHg [1,2,32]. Because most of the included trials did not report individual categorical transitions, the current evidence does not allow direct estimation of reclassification across AHA/ACC categories.

Pharmacological treatment remains the main therapeutic strategy for patients with persistent hypertension, high cardiovascular risk, comorbidities, or blood pressure values requiring larger and more controlled reductions [1,4,5,7,32,52]. The present findings do not support replacing antihypertensive medication with RT [11,15,16,17,18,53,54,55]. Most of the included studies involved medicated participants, stable pharmacological therapy, or mixed samples with different levels of pharmacological control. This improves clinical applicability but limits the attribution of blood pressure changes exclusively to RT. In medicated participants, part of the observed control may reflect baseline or concomitant pharmacological effects [1,4,7,8]. In non-medicated participants, the response may depend on baseline blood pressure, adherence, supervision, exercise intensity, intervention duration, and individual characteristics [13,17,18,19,53,54,55,56]. A medication-status sub-analysis was considered to isolate the effects of RT more clearly; however, the available trials did not provide sufficient stratified data for medicated and non-medicated participants. Therefore, this issue was addressed qualitatively and should be prioritised in future trials. RT should be interpreted as a complementary strategy within the comprehensive management of hypertension.

Medication use was an important source of clinical heterogeneity [1,4,7,11]. Some trials included participants receiving stable antihypertensive therapy, whereas others included mixed samples or provided limited information on medication type, dose, timing of administration or adherence [40,41,42,43,44,45,46,47,48]. This variability may attenuate, amplify or mask the independent effect of RT on blood pressure [1,4,7,8]. Future trials should standardise medication reporting, document medication changes during follow-up and analyse whether treatment status modifies the blood pressure response to RT [11,55,56,57,58].

Protocol heterogeneity was a central methodological limitation and should be considered when translating these findings into clinical practice. The included studies used dynamic RT, isometric handgrip training, elastic band training, and combined protocols [40,41,42,43,44,45,46,47,48]. Intensity was prescribed using %1RM [40,41,42,45,46,48], %MVC [43,45,47], subjective exertion scales [40,41,44,48], repetition maximum zones [45,46,48], elastic resistance, tolerance-based progression, or combinations of these methods [40,41,42,43,44,45,46,47,48]. Volume also varied in the number of exercises, sets, repetitions, contraction duration, rest intervals, weekly frequency, total intervention duration, and progression criteria [40,41,42,43,44,45,46,47,48]. These differences imply that the pooled estimate represents a general effect of RT-based interventions rather than a single reproducible prescription. Consequently, the findings support RT as a clinically useful complementary strategy, but they do not yet define an optimal dose-response model for adults with hypertension [15,16,17,18,53,54,55,56,59,60,61,62,63,64,65].

The subgroup analyses should also be interpreted with caution. Several subgroups included only one or a small number of studies, which reduces statistical power and increases the risk that apparent differences between RT modalities reflect sparse data, protocol variability or study-level characteristics rather than true comparative effects. Therefore, comparisons between dynamic RT, isometric handgrip training, elastic-band RT and combined protocols should be regarded as exploratory and hypothesis-generating rather than definitive evidence of superiority of one modality over another.

Overall, the findings support RT as an adjunctive intervention within the comprehensive management of hypertension. The main clinical benefit is related to partial reductions in SBP and DBP, a possible improvement in cardiovascular profile, and its contribution to a therapeutic lifestyle [6,11,12,13,14,15,16,17,18,53,54,55,56,59,60,61,62,63,64,65]. However, the current evidence does not confirm systematic reclassification across AHA/ACC blood pressure categories [32]. The certainty of the evidence was moderate for SBP and low to moderate for DBP, reflecting clinical heterogeneity, imputed dispersion values, variable medication control, and limited reporting of absolute changes in mmHg. Future randomised controlled trials should use standardised RT protocols, rigorous control and reporting of antihypertensive medication, absolute changes in mmHg, ambulatory and clinical blood pressure outcomes where possible, and individual-level analyses of category transitions. This design would allow a more precise estimation of the clinical relevance of RT in the treatment of hypertension.

The assessment of publication bias should be interpreted cautiously. Funnel plots, Begg’s test, Egger’s test, and trim-and-fill procedures have limited reliability when relatively few studies are available, and their interpretation is further restricted by differences in RT modality, outcome measurement, and pharmacological control. Consequently, the absence of clear funnel-plot asymmetry should not be interpreted as definitive evidence that publication bias was absent.

## 5. Conclusions

Resistance training significantly reduced SBP and DBP in adults with hypertension or elevated blood pressure. The effect was moderate for SBP and smaller for DBP, with moderate certainty for SBP and low-to-moderate certainty for DBP. These findings support RT as a complementary non-pharmacological strategy within the comprehensive management of hypertension.

The clinical relevance of these effects should be interpreted according to baseline blood pressure category, the magnitude of individual change, and AHA/ACC thresholds. The current evidence does not confirm systematic reclassification towards lower blood pressure categories. RT may contribute to blood pressure control and cardiovascular health, but it should complement, rather than replace, antihypertensive pharmacological therapy when medication is clinically indicated.

Heterogeneity in training modality, intensity, volume, frequency, duration, pharmacological control and blood pressure measurement limits reproducibility. The current evidence does not allow specific clinical prescriptions to be established with high certainty. Future randomised controlled trials should use standardised RT protocols, rigorous medication control, reporting of absolute blood pressure changes in mmHg and analyses of transitions across AHA/ACC categories to clarify the clinical usefulness of RT in the treatment of hypertension.

## Figures and Tables

**Table 1:** Participants, intervention, comparators, outcomes and study design (PICOS) criteria for the inclusion of randomised controlled trials.

Population	Hypertensive Adults (≥18 Years)
Intervention	Resistance Training (Dynamic resistance, isometric handgrip, elastic-band and combined resistance training)
Comparison	Resistance-training intervention group compared with another intervention group or a non-exercise control group
Outcomes	Systolic and diastolic blood pressure and blood pressure levels
Study design	Human randomized controlled trials

**Table 2:** Characteristics of the RCTs included in the current systematic review and meta-analysis.

Study	Sample, Sex and Age	Frequency/Duration	RT Modality	Exercise Intervention and Intensity	Pharmacological Control	BP Response
Abrahin et al. (2024) [40]	Total *n* = 52; RT: 27; CG: 25. Mixed; RT: 65.0 ± 5.0 y; CG: 67.3 ± 5.9 y.	3 d/wk; 12 wk	Dynamic RT	30–40 min/session; 2 sets of 6–10 reps; six resistance exercises; 90–120 s rest. Intensity: ≈70–85% 1RM + OMNI-RES; +2–5% load when 10 reps were completed in all sets during two consecutive sessions.	ARBs, ACE inhibitors, β-blockers, diuretics and CCB.	SBP: RT ↓, CG ↔. DBP: RT ↔, CG ↔.
Abrahin et al. (2022) [41]	Total *n* = 61; RT: 20; AT: 20; CG: 21. Mixed older adults; age not separated in extracted table.	3 d/wk; 12 wk; 6-wk washout	Dynamic RT	30–40 min/session; 2 sets of 6–10 reps; six resistance exercises; 90–120 s rest. Intensity: OMNI 7–8; load increased 2–10% after completion of 10 reps in all sets during two consecutive sessions.	ARBs, β-blockers, diuretics and CCB.	SBP: RT ↓, AT ↓, CG ↔. DBP: RT ↔, AT ↔, CG ↔.
Baghaiee et al. (2023) [42]	Total *n* = 40; RT: 20; CG: 20. Male; 45.3 ± 3.2 y.	3 d/wk; 12 wk	Dynamic RT	~40 min/session; 3 sets × 8 reps. Intensity: 80% 1RM; 1RM retested weekly and load adjusted to maintain 80% 1RM.	Participants under medical supervision and taking BP medication.	SBP: RT ↓, CG ↔/↑. DBP: RT ↓, CG ↔.
Cahu Rodrigues et al. (2019) [43]	Total *n* = 33; IHT and CG; responders analysed. Mixed; 67% female; 61 ± 2 y.	3 d/wk; 12 wk	Isometric handgrip	4 × 2-min contractions at 30% MVC, alternating hands, 1 min rest; home-based and supervised IHT merged. Intensity: 30% MVC; MVC reassessed at week 6.	All participants used antihypertensive medication; ARBs, ACE inhibitors, β-blockers, diuretics and CCB reported.	SBP: IHT ↓, CG ↓/↔. DBP: IHT ↓, CG ↔.
Son et al. (2020) [44]	Total *n* = 25; EX: 12; CON: 13. Female; EX: 67.8 ± 1.1 y; CON: 67.6 ± 1.3 y.	3 d/wk; 12 wk	Elastic-band RT	Total-body elastic-band programme including upper- and lower-limb exercises. Intensity progressed from 40–50% 1RM/RPE 11–12 to 60–70% 1RM/RPE 15–16.	Medication abstained ≥24 h before measurement; medication profile NR.	SBP: EX ↓, CON ↔. DBP: EX ↔, CON ↔.
Pedralli et al. (2020) [22]	Total *n* = 42; RT: 14; AT: 14; CT: 14. Mixed; 54 ± 11 y.	2 d/wk; 8 wk	Dynamic RT and combined training	RT: 6 exercises, 4 sets × 8–12 reps. CT: same RT exercises at 2 sets × 12 reps + 20 min aerobic cycling. Intensity: 60–80% 1RM; aerobic component 50–75% HR reserve.	Antihypertensive medication profile NR; β-blocker adjustment through RPE for aerobic prescription.	SBP: RT ↓, AT ↓, CT ↔. DBP: RT ↔, AT ↔, CT ↓.
Boeno et al. (2020) [24]	Total *n* = 42; RT: 15; AT: 15; CON: 12. Mixed; RT: 46.1 ± 7.2 y; AT: 45.8 ± 6.8 y; CON: 44.3 ± 8.3 y.	3 d/wk; 12 wk	Dynamic RT	2–3 sets of multi-joint and single-joint resistance exercises; 120 s rest. Intensity progressed through RM zones: 15–20, 10–15 and 8–12 reps; volume increased from 2 to 3 sets.	At least one antihypertensive medication; ARB, ACEI, β-blocker, diuretic and CCB combinations reported.	SBP: RT ↓, AT ↓, CON ↔/↑. DBP: RT ↔/↓, AT ↔, CON ↔.
Fecchio et al. (2023) [21]	Total *n* = 62; DRT: 16; IHT: 15; CRT: 15; CON: 16. Male; 50–55 y across groups.	3 d/wk; 10 wk	Dynamic RT, IHT and combined RT	DRT: 8 exercises, 3 sets to moderate fatigue. IHT: 4 × 2 min at 30% MVC. CRT: DRT + IHT. Intensity: DRT began at 50% 1RM with load progression; IHT maintained at 30% MVC.	Treated hypertension; monotherapy/polytherapy; ARB, ACEi, CCB, diuretics and statins reported.	SBP: DRT ↓, IHT ↔, CRT ↓, CON ↔. DBP: DRT ↔, IHT ↔, CRT ↔, CON ↔.
Silva de Sousa et al. (2024) [45]	Total *n* = 59; DRT/IHT/CRT/CON. Male; middle-aged men; exact group ages NR in accepted extract.	3 d/wk; 10 wk	Dynamic RT, IHT and combined RT	Same parent trial as Fecchio et al.; DRT at 50% 1RM; IHT at 30% MVC; CRT = DRT + IHT; CON stretching. Outcome: ambulatory BP.	Treated hypertension; details from parent trial.	24-h/awake/asleep SBP and DBP: ↔ all groups.
Alemayehu (2023) [46]	Total *n* = 46; ATG: 11; RTG: 11; CTG: 12; CG: 12. Male; 45.28 ± 7.44 y.	12 wk	Dynamic RT and combined training	Circuit RT using upper- and lower-limb exercises, 3 × 10 reps; CT used 5 RT exercises × 2 sets + aerobic training. Intensity: Borg RPE 11–13; aerobic 64–76% HRmax, not exceeding 80%.	BP medication timing controlled before measurements; medication types NR.	SBP: RT ↓, CT ↓, AT ↓, CG ↔. DBP: RT ↓, CT ↓, AT ↓, CG ↔.
Ankit Kumar et al. (2025) [47]	Total *n* = 40; Aerobic: 10; IHT: 10; Yoga: 10; CG: 10. Mixed; >18 y; exact mean age NR.	4 d/wk; 6 wk	Isometric handgrip	IHT: 4 × 2-min handgrip contractions at 30% MVC with 3-min rest. Intensity maintained at 30% MVC with feedback and encouragement.	All participants continued antihypertensive medications.	SBP: IHT ↔, Aerobic ↓, Yoga ↓, CG ↔. DBP: IHT ↔, Aerobic ↓, Yoga ↓, CG ↔/↑.
Caminiti et al. (2021) [48]	Total n = 36; ACE: 12; HIIE: 12; CE: 12. Male; 64.5–66 y across groups.	Acute testing before/after 12 wk ACT	Combined aerobic + RT acute session	CE: 25 min treadmill at 55–70% VO2peak + resistance exercises, 2 × 10 reps at 60% 1RM. Chronic training was aerobic continuous training, not isolated RT.	Stable pharmacological treatment; ACEi/ARBs, CCB, β-blockers and diuretics reported.	Acute PEH: ↓. Chronic isolated RT effect: NR.

Abbreviations: RCTs: randomized controlled trials; RT: resistance training; DRT: dynamic resistance training; IHT: isometric handgrip training; CRT: combined resistance training; AT: aerobic training; CT: combined training; CON/CG: control group; SBP: systolic blood pressure; DBP: diastolic blood pressure; BP: blood pressure; HR: heart rate; HRmax: maximal heart rate; 1RM: one-repetition maximum; MVC: maximal voluntary contraction; RPE: rating of perceived exertion; ARB: angiotensin receptor blocker; ACEi: angiotensin-converting enzyme inhibitor; CCB: calcium channel blocker; NR: not reported; PEH: post-exercise hypotension; ↓: decrease; ↔: no relevant change; ↑: increase.

**Table 3:** Certainty of evidence for the primary outcomes using the GRADE approach.

Outcome	Summary of Effect	Certainty (GRADE) and Rationale
SBP	SMD = −0.77 (95% CI: −1.06 to −0.48); I^2^ = 35%; 12 RCTs.	Moderate. Downgraded for clinical heterogeneity, imputed dispersion values in some studies, and variability in RT prescription and medication control.
DBP	SMD = −0.43 (95% CI: −0.67 to −0.19); I^2^ = 0%; 12 RCTs.	Low-to-moderate. Downgraded for smaller effect magnitude, imprecision in some comparisons, and variability in measurement and pharmacological control.

Abbreviations: SBP, systolic blood pressure; DBP, diastolic blood pressure; SMD, standardised mean difference; CI, confidence interval; RCTs, randomised controlled trials.

## Data Availability

The original contributions presented in the study are included in the article; further inquiries can be directed to the corresponding authors.

## Supplementary Materials

The following supporting information can be downloaded at: https://www.mdpi.com/article/10.3390/jcm15145531/s1, Table S1: PRISMA 2020 checklist [31].
